# Exploring medical food supplementation as an adjunct strategy for microbiota restoration and seizure reduction in CDKL5 deficiency disorder

**DOI:** 10.1002/epi4.70369

**Published:** 2026-10-13

**Authors:** Elisa Borghi, Matteo Domenico Marsiglia, Francesca Triva, Emerenziana Ottaviano, Ilaria Viganò, Miriam Nella Savini, Valentino Costabile, Aglaia Vignoli

**Affiliations:** ^1^ Department of Health Sciences Università degli Studi di Milano Milan Italy; ^2^ Unit of Microbiology and Virology ASST Santi Paolo e Carlo Milan Italy; ^3^ Child Neurology – Epilepsy Centre ASST Santi Paolo Carlo, Member of ERN‐Epicare Milan Italy; ^4^ Childhood Neurology and Psychiatry Unit Azienda Socio Sanitaria Territoriale Grande Ospedale Metropolitano (ASST GOM) Niguarda Milan Italy

**Keywords:** butyrate, cdkl5, epilepsy, microbiota‐gut‐brain axis

## Abstract

**Objectives:**

CDKL5 deficiency disorder (CDD) is a developmental and epileptic encephalopathy characterized by early‐onset, treatment‐resistant seizures and severe neurodevelopmental impairment. Increasing evidence suggests that alterations in the gut–brain axis may contribute to disease pathophysiology, raising interest in microbiota‐targeted interventions. In this study, we evaluated the effects of a prebiotic‐based nutritional intervention on clinical outcomes, gut microbiota composition, and fatty acid profiles in individuals with CDD.

**Methods:**

Thirteen participants with drug‐resistant epilepsy were enrolled (one male; median age, 12 years [IQR, 8.8–17.5]), and ten completed the longitudinal intervention protocol. The protocol comprised two medical‐food interventions separated by washout periods: MF1 contained alpha‐lactalbumin, inulin, and fructo‐oligosaccharides, whereas MF2 contained the MF1 formulation supplemented with sodium butyrate. Clinical assessments, gut microbiota profiling, and fecal and systemic fatty acid measurements were performed at predefined study time points.

**Results:**

Seizure response varied substantially between participants. Two subjects showed a 52% and 57% reduction in monthly seizure frequency during MF1, whereas two different participants experienced reductions greater than 90% during MF2 (94% and 100%, respectively). At the cohort level, the lowest median seizure frequency was observed at the final study time point. A progressive decrease in CDD severity scale scores was also observed over the course of the study.

Intervention phases coincided with transient enrichment of short‐chain fatty acid (SCFA)‐producing genera, including *Faecalibacterium, Blautia*, and *Dorea*. Microbiota composition differed between responders and non‐responders: higher seizure burden was linked to increased *Veillonella*, whereas lower seizure frequency corresponded to enrichment of *Dialister* and *Gemmiger*. Fecal and systemic SCFA concentrations generally decreased during intervention phases, regardless of clinical response.

**Significance:**

Overall, these findings support a possible interaction between nutritional supplementation, microbial dynamics, and clinical manifestations in CDD. Larger studies will be necessary to clarify the therapeutic relevance of personalized microbiota‐targeted approaches in CDD‐related epilepsy.

**Plain Language Summary:**

CDKL5 deficiency disorder (CDD) is a rare condition that causes severe developmental difficulties and seizures that are difficult to control. We studied whether two medical food interventions targeting the gut microbiota, one of which also contained sodium butyrate, could influence seizures, disease severity, gut microbiota composition, and metabolite production in people with CDD. Some participants experienced substantial reductions in seizure frequency alongside changes in gut bacteria, although responses varied considerably between individuals. These preliminary findings suggest that microbiota‐targeted, non‐pharmacological approaches may have potential as supportive strategies in CDD, but larger studies are needed to confirm these observations.

**Key points**

1. Medical food supplementation was associated with reduced seizure frequency in a subset of individuals with CDD.
2. Medical food supplementation was associated with transient changes in gut microbiota composition.
3. Responders and non‐responders showed distinct gut microbial profiles associated with seizure burden.

## 1 INTRODUCTION

In recent years, increasing attention has been directed toward the gut–brain axis, a bidirectional communication system linking intestinal and neurological function.^1^ Alterations in gut microbiota composition have been associated with several neurological disorders, raising interest in how dietary interventions may influence disease progression through microbiota modulation.^2^, ^3^

Among the metabolites produced by intestinal bacteria, butyrate has received particular attention. This short‐chain fatty acid, generated through microbial fermentation of dietary fibers, is involved in several processes relevant to neurological health, including immune regulation, inflammatory control, epigenetic modulation, and neuroinflammatory responses.^4^ These observations have encouraged the investigation of microbiota‐targeted approaches, such as prebiotics, probiotics, and ketogenic dietary interventions, particularly in neurological conditions associated with drug‐resistant epilepsy.^2^, ^3^

Cyclin‐dependent kinase‐like 5 (CDKL5) deficiency disorder (CDD) is a rare X‐linked neurodevelopmental condition characterized by early‐onset refractory epilepsy, severe developmental impairment, and frequent gastrointestinal disturbances.^3^, ^5^ Emerging evidence also suggests that gut microbial alterations may contribute to symptom severity in CDD, supporting the hypothesis that the gut–brain axis could represent a relevant therapeutic target.^6^, ^7^, ^8^ Based on this rationale, the present study evaluates the effects of two medical foods, one supplemented with butyrate and one without butyrate enrichment, on clinical parameters, gut microbiota composition, and metabolic profiles in individuals with CDD.

## 2 METHODS

### 2.1 Study design and intervention

The effects of two medical foods (MF1 and MF2; Kolfarma s.r.l.) containing alpha‐lactalbumin (ALAC), inulin, and fructooligosaccharides (FOS), with (MF2) or without (MF1) sodium butyrate (NaB), were evaluated in children and adults with CDKL5 deficiency disorder (CDD) in a single‐arm, open‐label trial^9^ (NCT06448663). MF1 was administered at a dose of one sachet/day, providing a daily intake of 500 mg ALAC, 375 mg inulin, and 375 mg FOS. MF2 dosing was weight‐adjusted: participants weighing ≤ 30 kg received one sachet/day, providing 1000 mg ALAC, 400 mg inulin, 375 mg FOS, and 625 mg NaB daily, whereas participants weighing > 30 kg received one sachet twice daily, corresponding to a total daily intake of 2000 mg ALAC, 800 mg inulin, 750 mg FOS, and 1250 mg NaB. The primary endpoint was seizure response, defined as a reduction in monthly seizure frequency of at least 50%, 75%, or complete seizure freedom (100%) compared with baseline. To this end, seizure frequency was prospectively documented in seizure diaries completed by the parents/caregivers. Seizure counts were subsequently extracted from these diaries by the staff and transcribed into the study database for analysis.

The study included a 12‐week MF1 supplementation phase, a 4‐week washout, a 12‐week MF2 phase, and a final 4‐week washout. Clinical evaluations were performed at baseline, after MF1, and at the end of treatment. Secondary endpoints included changes in sleep (SDSC scale), behavior (RSBQ), gastrointestinal symptoms (GISI), disease severity (CDD severity scale), motor function (MBAS), and global clinical improvement (CGI‐C).

Caregivers collected stool samples (eNAT®, Copan), dried blood spots, and filled the Bristol scale chart and 3‐day dietary records (including one weekend day) at scheduled time points. Dietary intake was analyzed using MètaDieta® software (METEDA srl, Italy).

The study protocol, amendments, and informed consent were approved by the Comitato Etico Territoriale 3 (CET 3), ASST Grande Ospedale Metropolitano Niguarda, Milan, Italy (approval no. 4189_17.04.2024_N_bis). The study was conducted in accordance with local regulations and institutional requirements. Written informed consent was obtained from participants' legal guardians or next of kin.

### 2.2 Participants and eligibility criteria

Children and adults aged 3–50 years (≥ 12 kg) with a clinical diagnosis of CDKL5 deficiency disorder (CDD) and a pathogenic *CDKL5* variant were eligible. Participants were recruited via physician or caregiver referral through the CDKL5 Patients Association (CDKL5 Insieme verso la cura). Only individuals with drug‐resistant seizures on a stable antiepileptic regimen for at least 4 weeks were included. No changes were made to concomitant antiseizure medications (ASMs) or other non‐pharmacological therapies during the study period. Written informed consent was obtained from parents, legal guardians, or authorized representatives.

Exclusion criteria included participation in another clinical trial, organic gastrointestinal disorders (e.g., food allergies, celiac disease), special diets, PEG use, and antibiotic or probiotic use within the month prior to enrollment.

### 2.3 Microbiota characterization

Microbial DNA was extracted from fecal samples using a Maxwell® RSC Fecal Microbiome DNA Kit and the Maxwell instrument from Promega, following the manufacturer's instructions. Amplicon libraries were generated targeting the V3–V4 hypervariable regions of the bacterial 16S rRNA gene using the Quick‐16S™ NGS Library Prep Kit (Zymo Research), according to the manufacturer's protocol. Sequencing was performed on an Illumina MiSeq platform.

Bioinformatic analyses were performed as described in Riva et al.^10^ Briefly, raw sequencing reads were processed using QIIME 2 (version 2025.4). Demultiplexed paired‐end reads were quality‐filtered, trimmed, and denoised using the DADA2 plugin implemented in QIIME 2 to infer amplicon sequence variants (ASVs). A phylogenetic tree was constructed from the inferred ASV sequences and used for downstream phylogeny‐based analyses. Taxonomic assignment was performed by training a Bayesian classifier (using sklearn 1.4.2 implemented in QIIME2) with the full‐length sequences from the Genome Taxonomy Database (GTDB) r220,^11^ following the extraction of the target 16S rRNA region, guided by the forward and reverse primer sequences.

Alpha diversity indices, including Pielou's evenness, Chao1 richness, Shannon index, Faith's phylogenetic diversity, and observed species, were calculated to assess within‐sample diversity. Beta diversity was evaluated using unweighted and weighted UniFrac distance metrics and visualized by principal coordinates analysis (PCoA).^12^ Relative abundances were calculated at the genus level for compositional analyses.

The 16S rRNA gene sequencing data generated in this study are available in the NCBI Sequence Read Archive (SRA) under submission accession ID SUB16206286.

### 2.4 Fecal and systemic fatty acids analysis

Fatty acids in fecal samples and dried blood spots (DBS) were quantified using a targeted metabolomic approach according to a previously described analytical workflow.^13^

For fecal analysis, samples were first acidified and extracted with organic solvents in the presence of internal standards. Quantification of acetate, propionate, isobutyrate, butyrate, 2‐methylbutyrate, isovalerate, valerate, caproate, caprylate, and caprate was then performed by gas chromatography–mass spectrometry (GC–MS).

Systemic fatty acid profiling was carried out on DBS samples. After protein precipitation, samples underwent chemical derivatization with internal standards prior to liquid chromatography–tandem mass spectrometry (LC–MS/MS) analysis. The panel included propionate, isobutyrate, butyrate, isovalerate, valerate, caproate, caprylate, caprate, and laurate.

Quantification was based on calibration curves, and blank sample values were subtracted from all measurements to improve analytical accuracy.

### 2.5 Statistical analysis

Clinical variables measured at baseline and at the end of follow‐up were compared using the Wilcoxon signed‐rank test. Variables collected across three time points were instead analyzed with the Friedman test for repeated measures.

Associations between genus‐level microbial abundance and clinical parameters, including seizure frequency, gastrointestinal symptoms, sleep disturbances, CDD severity, and CGI scores, were evaluated using MaAsLin2 with linear mixed‐effects models. For seizure‐related analyses, only taxa detected in at least 10% of patients and identified at a minimum of two time points were retained. For the remaining clinical variables, taxa had to be present at two or more time points in at least 50% of samples to be included in the analysis.

Before modeling, count tables were normalized using total‐sum scaling and subsequently log‐transformed. *p*‐values were corrected according to the Benjamini–Hochberg false discovery rate (FDR) method. Taxa with *q*‐values < 0.05 were considered statistically significant, whereas taxa with *p* < 0.05 but *q* ≥ 0.05 were treated as nominally significant and maintained for visualization purposes.

To further investigate treatment‐related effects, taxa abundance at each treatment time point was compared with baseline values using paired Wilcoxon tests. All statistical analyses were conducted in RStudio.

## 3 RESULTS

### 3.1 Clinical characteristics of the study cohort

Thirteen subjects were enrolled in the study, with a median age of 12 years (IQR 8.8–17.5). All participants had epilepsy, and seizure onset generally occurred within the first six months of life. In most cases (69.2%), seizures began between 3 and 6 months of age, whereas onset before 3 months and after 6 months was reported in 15.3% of subjects each.

Most patients required multiple antiseizure medications (ASMs): 53.8% were receiving three or more ASMs, 38.4% were treated with two drugs, and only one subject (7.6%) was on monotherapy. Weekly seizures represented the most common seizure pattern (69.2%), while daily seizures were observed in 23.0% of the cohort.

Motor stereotypies and autonomic disturbances were present in all subjects. Sleep disorders were also highly prevalent, affecting 84.6% of participants. Visual impairment was reported in 30.7% of cases, behavioral impairment in 69.2%, and gastrointestinal symptoms in 46.1%.

Baseline clinical characteristics of the CDD cohort are summarized in the Table S1.

Among the 13 subjects initially characterized, 10 (one male, median age (IQR), 12 [8.25–20.5]) completed the longitudinal nutritional intervention and were therefore included in the downstream analyses. Overall, 50 stool samples and 30 DBS cards were collected across the study time points according to the experimental design and were included in the microbiota and metabolomic analyses, respectively.

Three participants withdrew from the study: one due to diarrhea, one due to constipation, and one following the initiation of antibiotic therapy for a respiratory illness.

Descriptive statistics across the different study time points are presented in Table 1.

**TABLE 1:** Clinical characteristics of CDKL5 subjects (*n* = 10) during the study period.

Clinical feature	t0	t1 (MF1)	t2	t3 (MF2)	t4	*p*
Seizure frequency per month, median (IQR)	20.8 (12–56)	25.2 (11.3–61)	32 (5–66)	31.2 (19–61.3)	14 (8–44)	0.278
CDD severity scale, mean ± SD	46.1 ± 8.6	/	/	/	40.8 ± 8.5	**0.015**
CDKL5 developmental score (CDS), median (IQR)	2.5 (2–3)	/	/	/	2 (2–3)	0.484
Motor‐Behavioral Assessment Scale (MBAS), mean ± SD	57.3 ± 17.4	/	/	/	55.7 ± 14.4	0.859
RTT behavior questionnaire (RSBQ), mean ± SD	35.9 ± 13.7	/	/	/	38.2 ± 13.6	0.406
Gastrointestinal Severity Index (GISI) scale, median (IQR)	2.5 (1–4)	/	1.5 (1–3)	/	1 (0–3)	0.216
Sleep Disturbance Scale for Children (SDSC), mean ± SD	52.6 ± 14.7	/	50.0 ± 11.5	/	49.5 ± 13.8	0.349
Clinical global impression‐ improvement scale (CGI‐I), median (IQR)	/	/	3 (3–4)	/	3 (2–4)	0.776
Bristol Stool Scale, median (IQR)	3 (2–4)	3 (2–3)	3 (3–4)	3.5 (3–4)	2 (2–3)	0.292

*Note*: Values were reported according to data distribution. Variables with a normal distribution are presented as mean ± standard deviation, whereas non‐normally distributed variables are expressed as median and interquartile range (IQR). Bold value indicates statistical significance.

The primary endpoint was the response of drug‐resistant seizures during the two 12‐week treatment periods, defined as a reduction of ≥ 50%, ≥ 75%, or complete seizure remission (100%) compared with baseline monthly seizure frequency. Seizure frequency varied considerably between subjects, with fluctuating median values across intervention and washout phases. The lowest median seizure frequency was recorded at the final study time point. Individual responses were heterogeneous. During the first intervention phase (MF1), two participants experienced a 52% and 57% reduction in seizure frequency relative to baseline. In the second intervention phase (MF2), one subject showed a 94% reduction in seizure frequency, while another achieved seizure freedom.

The CDD severity scale showed a clearer temporal pattern, with scores decreasing from baseline (t0) to the final time point (t4). This reduction was observed at the cohort level and was accompanied by comparable distributions across subjects, indicating a shift toward lower severity scores at the final assessment (*p* = 0.015).

### 3.2 Longitudinal microbiome profiling

Ten individuals with CDD who completed the study were longitudinally followed across five time points (t0–t4). Dietary intake was monitored throughout, with no significant changes in energy, macronutrient, or fiber intake across time points (Table S2).

**FIGURE 1:**
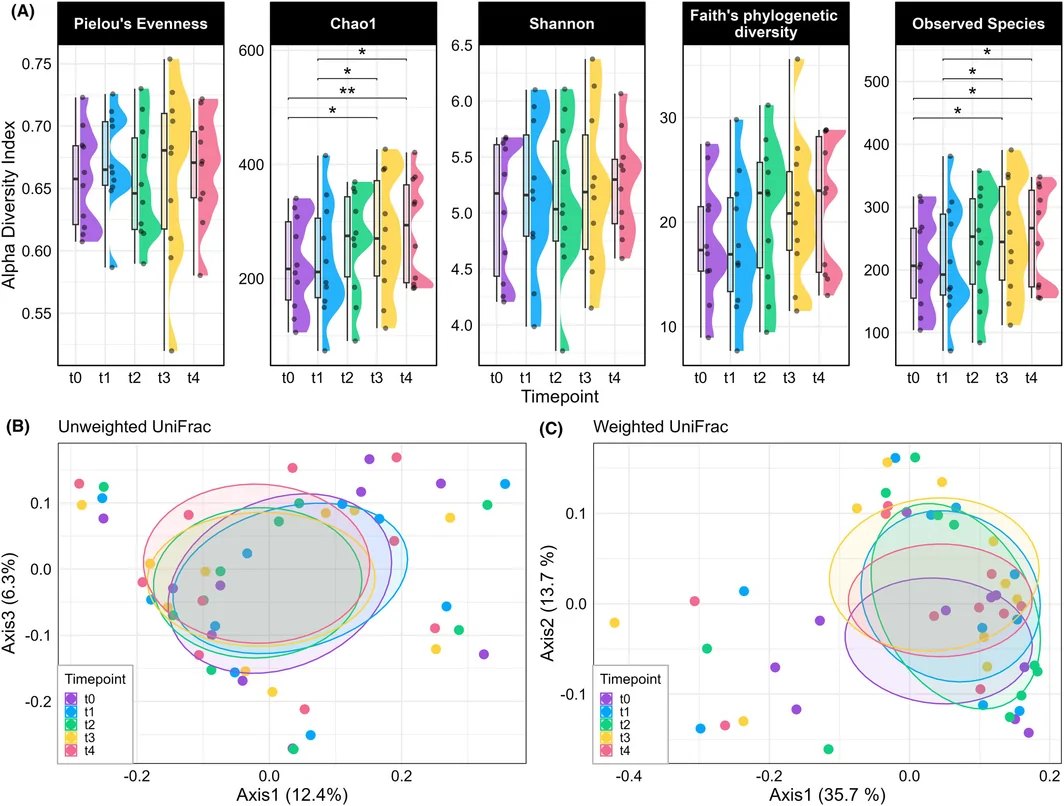
Longitudinal assessment of gut microbiota diversity across the study time points (t0–t4). (A) Raincloud plots show alpha diversity metrics, including Pielou's evenness, Chao1 richness, Shannon index, Faith's phylogenetic diversity, and observed species for each time point. (B) Principal coordinates analysis (PCoA) based on unweighted UniFrac distances and (C) weighted UniFrac distances. t0 corresponds to baseline (purple), t1 to MF1 nutritional intervention (blue), t2 to the subsequent washout period (green), t3 to MF2 nutritional intervention (yellow), and t4 to the final washout (red). *p < 0.05; **p < 0.01; ***p < 0.001.

Alpha diversity analyses (Figure 1A) indicated that community evenness and overall diversity remained relatively stable throughout the study, as reflected by Pielou's evenness and Shannon index values (*p* > 0.05). By contrast, microbial richness increased over time. Both observed species counts and Chao1 indices were significantly higher at later study time points, particularly during the second intervention and washout phases (t3–t4). Faith's phylogenetic diversity followed a similar pattern, although statistical significance was not maintained after correction for multiple testing. Community composition was further explored using unweighted and weighted UniFrac distance metrics (Figure 1B). Principal coordinate analysis (PCoA) based on unweighted UniFrac distances showed substantial overlap among time points, suggesting that the core phylogenetic structure of the microbiota remained generally stable over the course of the study. Weighted UniFrac analysis, however, revealed a partial temporal separation according to the intervention phase. The clearest shift was observed during the MF2 intervention (t3), followed by a partial return toward baseline configuration during the second washout period (t4).

To summarize compositional dynamics, we assessed the relative abundance of the most prevalent bacterial genera across time points (Figure 2A). Overall, genus‐level profiles remained largely stable, with no marked expansion or depletion at the cohort level. Nonetheless, gradual shifts emerged during intervention phases. At t3, *Faecalibacterium* showed a modest increase (0.018 vs. 0.045; *p* = 0.168), while *Prevotella* (0.061 vs. 0.024; *p* = 1.000) and *Bacteroides* (0.25 vs. 0.13; *p* = 0.002) decreased relative to t0. During the second washout, partial re‐equilibration was observed, with *Faecalibacterium* declining (0.028; *p* = 0.771) and *Bacteroides* increasing (0.152; *p* = 0.194).

**FIGURE 2:**
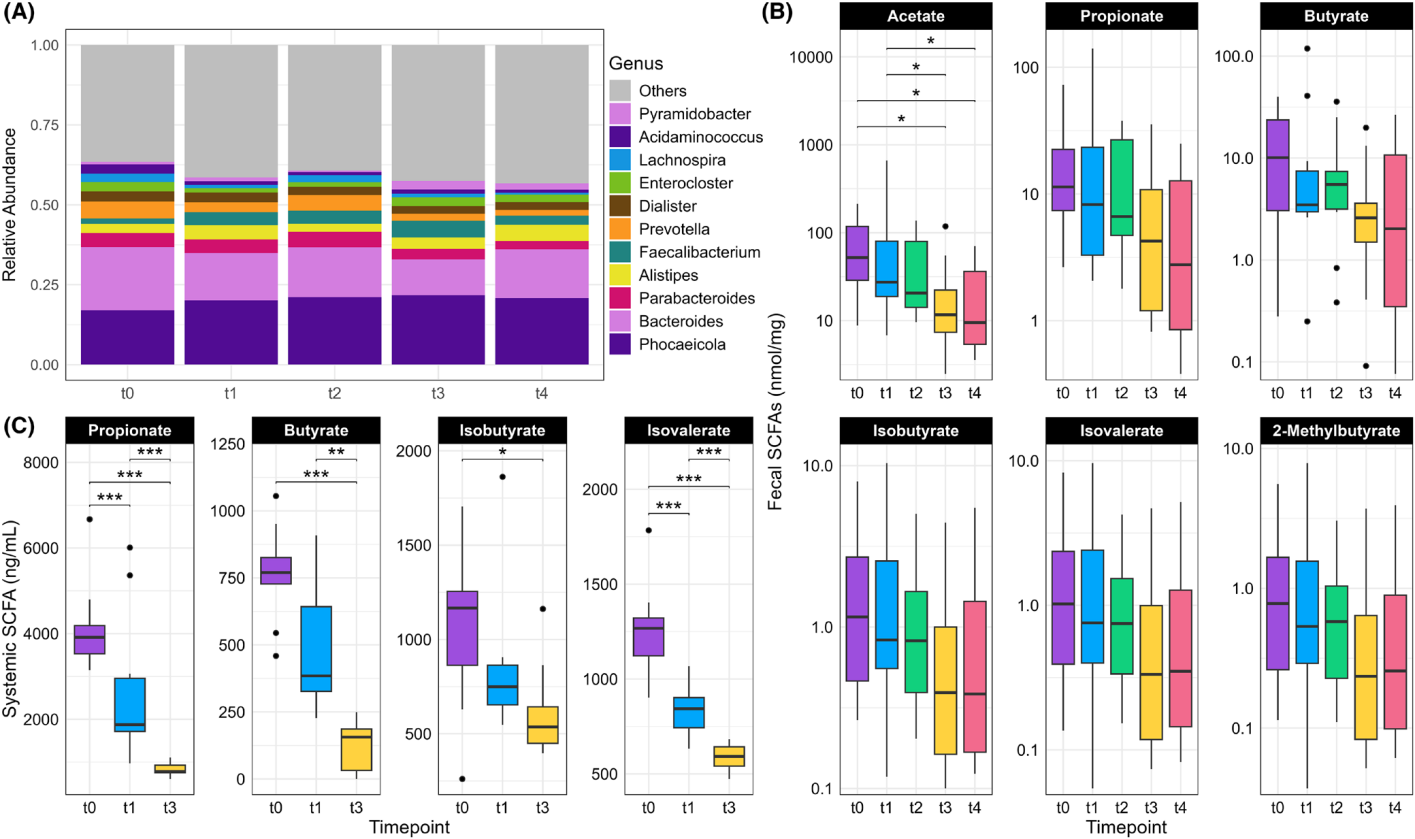
Integrated overview of gut microbial composition and short‐chain fatty acid (SCFA) profiles across study time points. (A) Relative abundance of the most prevalent bacterial genera at each time point. (B) Fecal concentrations of SCFAs, including acetate, propionate, butyrate, isobutyrate, isovalerate, and 2‐methylbutyrate. Baseline (t0, purple), MF1 nutritional intervention (t1, blue), first washout period (t2, green), MF2 nutritional intervention (t3, yellow), and second washout period (t4, red). (C) Dried blood spot concentrations of selected SCFAs (propionate, butyrate, isobutyrate, and isovalerate), shown as boxplots across t0, t1, and t3. *p < 0.05; **p < 0.01; ***p < 0.001.

Fecal fatty acid concentrations were then evaluated to investigate changes in microbial metabolic activity (Figure 2B; Supporting Information S3). At t1, short‐chain fatty acid (SCFA) levels displayed a modest overall reduction. A more pronounced decrease emerged during the butyrate‐supplemented intervention (t3), particularly for acetate, whose levels were significantly lower compared with both baseline (*p* = 0.017) and t1 (*p* = 0.035). This reduction remained evident during the following washout phase (t4; vs. t0: *p* = 0.017; vs. t1: *p* = 0.034). Propionate, butyrate, and branched‐chain fatty acids (BCFAs) also tended to decrease during t3, although these variations did not reach statistical significance.

To explore whether these microbiota‐related metabolic changes were reflected systemically, circulating fatty acids were quantified in dried blood spots collected at selected time points (Figure 2C; Supporting Information S4). Overall, systemic SCFA profiles paralleled the trends observed in fecal samples.

### 3.3 Gut microbiome and seizure frequency (primary outcome)

To examine inter‐individual variability in treatment response, participants were stratified as responders or non‐responders based on seizure frequency reduction during the intervention phases (t1 and t3). Responders (> 50% reduction in monthly seizures; *n* = 2) were compared to non‐responders (*n* = 8). No statistical analyses were performed due to the small sample size.

At the compositional level, genus‐level profiles differed between groups at baseline and during the first intervention (Figure 3A). Responders showed a distinct microbial composition with shifts from t0 to t1, including increased *Faecalibacterium* and *Phocaeicola* and decreased *Bacteroides* and *Prevotella*. In contrast, non‐responders exhibited a more stable genus‐level structure across time points.

**FIGURE 3:**
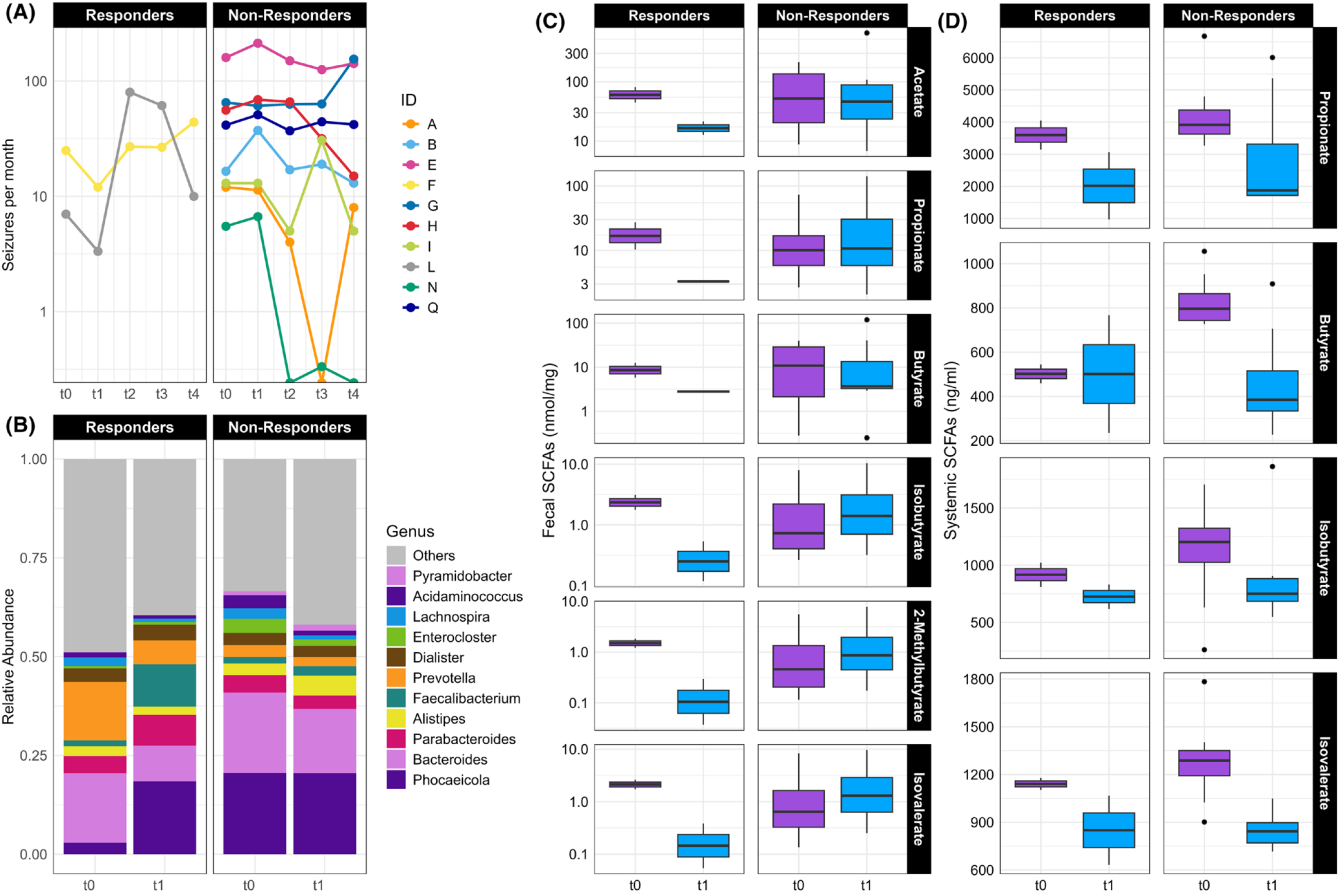
Gut microbiota composition and SCFAs profiles stratified by seizure response during the first nutritional intervention. Subjects were stratified into responders and non‐responders based on changes in seizure frequency between baseline (t0) and the intervention time point (t1). (A) Seizure frequency during the MF1 supplementation. (B) Genus‐level relative abundance profiles at t0 and t1 in responders and non‐responders, shown as stacked bar plots. Only the most abundant genera are displayed, with low‐abundance taxa grouped as “Others”. (C) Fecal SCFA concentrations at t0 and t1, including acetate, propionate, butyrate, and branched‐chain fatty acids. (D) Systemic SCFA concentrations measured in DBS at t0 and t1.

Differences were also observed in microbial metabolic outputs. In responders, fecal SCFA concentrations (Figure 3B) decreased at t1 relative to baseline, including acetate, propionate, butyrate, and BCFAs. Non‐responders showed greater variability, with overlapping SCFA distributions between t0 and t1.

Systemic SCFA profiles showed similar group differences (Figure 3C). In responders, circulating SCFAs generally declined from baseline to intervention, except for butyrate, whereas non‐responders exhibited a consistent decrease across all SCFAs.

To evaluate microbiota differences during the second intervention, participants were stratified as responders (*n* = 2) and non‐responders (*n* = 8) based on seizure frequency changes between t0 and t3 (Figure 4). At the genus level, distinct compositional profiles were observed between groups at both t2 and t3 (Figure 4A). Responders exhibited noticeable shifts in dominant genera during the intervention, including *Faecalibacterium, Bacteroides, Lachnospira*, and *Pyramidobacter*, whereas non‐responders showed a more stable microbial structure.

**FIGURE 4:**
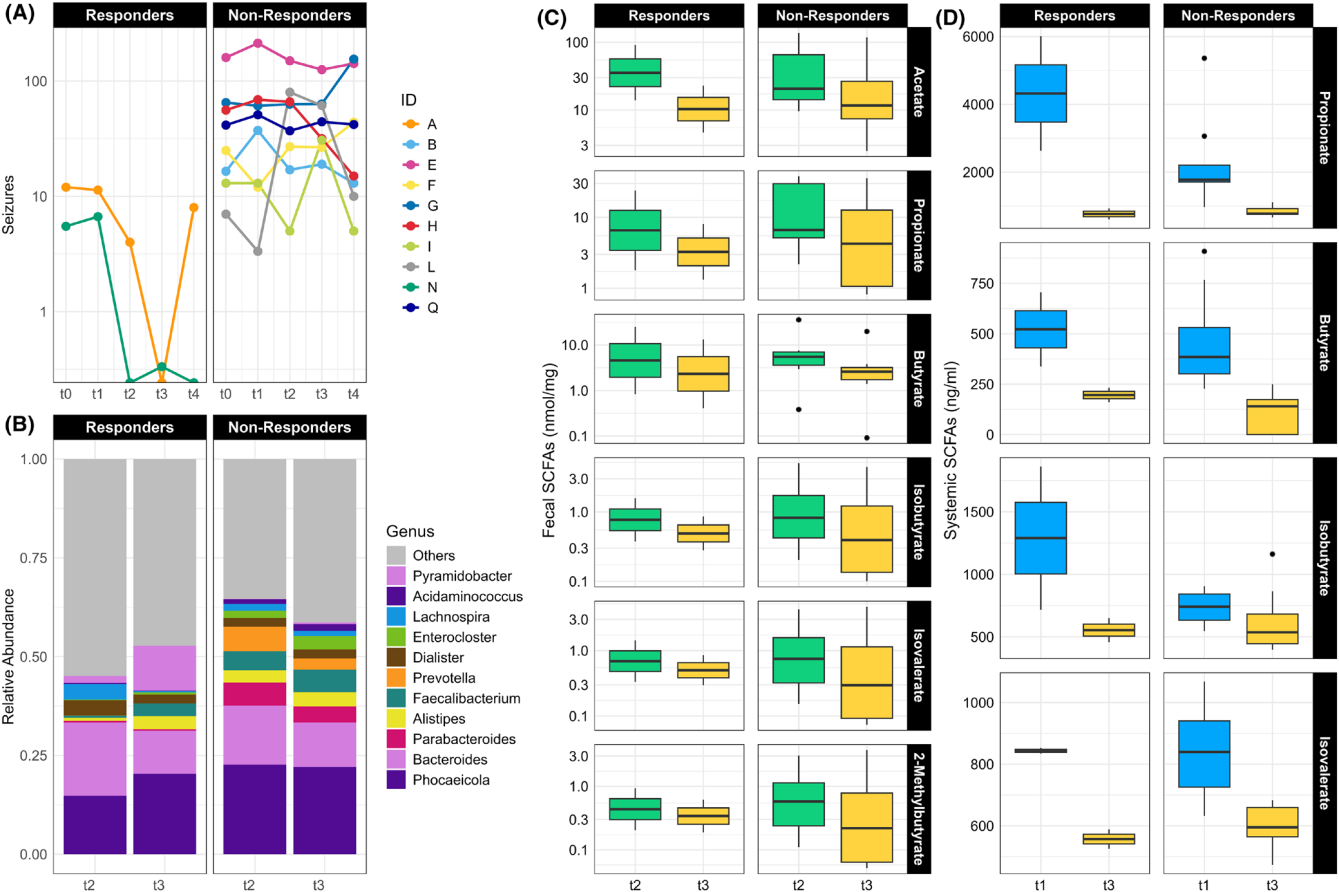
Gut microbiota composition and SCFAs profiles stratified by seizure response during the second nutritional intervention. Subjects were stratified into responders and non‐responders based on changes in seizure frequency between baseline (t0) and the intervention time point (t3). (A) Seizure frequency during the MF2 supplementation. (B) Genus‐level relative abundance profiles at t2 and t3 in responders and non‐responders, shown as stacked bar plots. Only the most abundant genera are displayed, with low‐abundance taxa grouped as “Others”. (C) Fecal SCFA concentrations at t2 and t3, including acetate, propionate, butyrate, and branched‐chain fatty acids. (D) Systemic SCFA concentrations measured in DBS at t1 and t3.

In contrast, SCFA profiles changed similarly in both groups (Figure 4B,C). Fecal SCFA concentrations decreased from t2 to t3 across all measured metabolites, including acetate, propionate, butyrate, and BCFAs. Systemic SCFAs showed a comparable decline, with circulating levels decreasing from t1 to t3 in both responders and non‐responders.

Longitudinal microbiota dynamics were assessed through pairwise comparisons between adjacent time points, highlighting temporal shifts in taxon abundance (Supporting Information S5). Treatment phases were associated with transient increases in genera such as *Faecalibacterium, Blautia*, and *Dorea*, while washout periods showed enrichment of taxa including *Bacteroides* and *Prevotella*.

Exploratory association analysis further identified taxa linked to seizure frequency (Figure 5A), with several butyrate‐producing genera inversely associated and taxa such as *Veillonell*a and *Enterobacteriaceae* positively associated.

**FIGURE 5:**
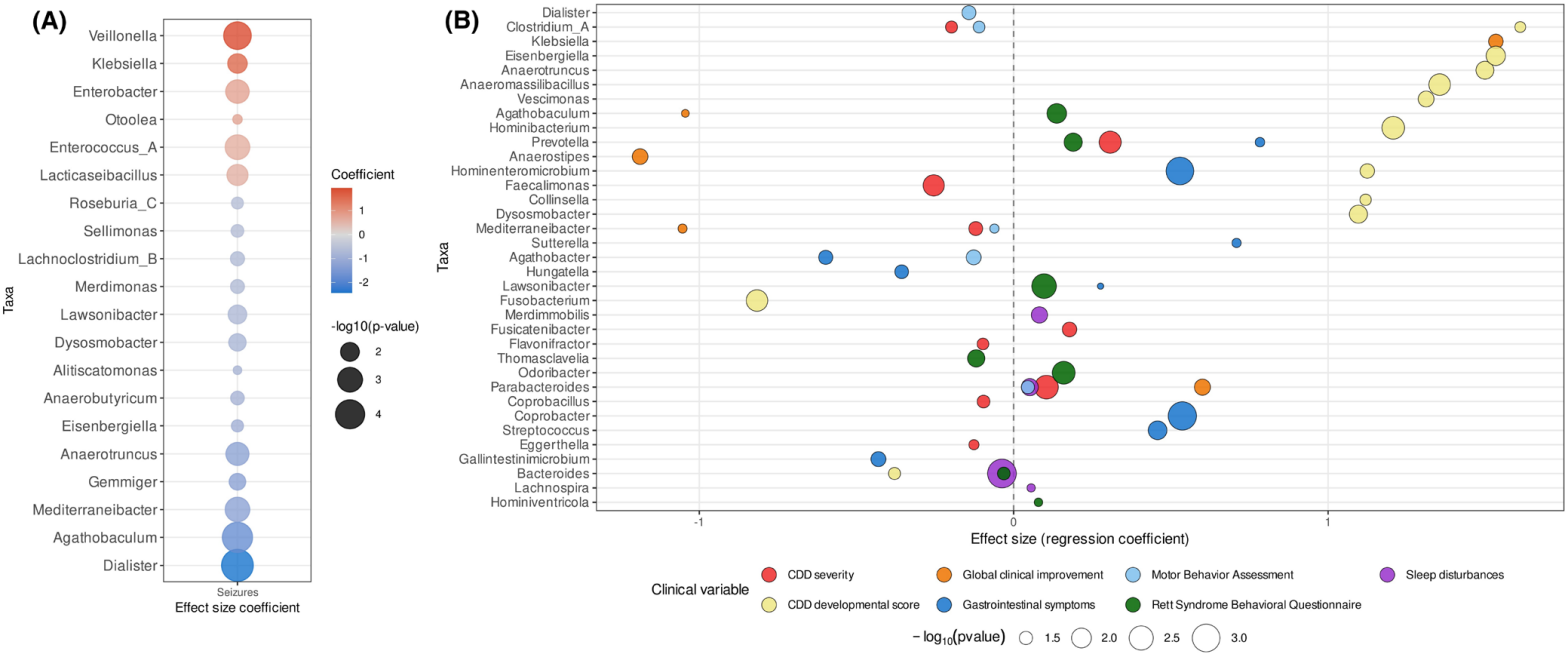
Bubble plot showing the effect size coefficients (*x*‐axis) of genus‐level taxa (*y*‐axis) identified as nominally significant across the investigated clinical variables. (A) Bubble plot showing the significance (dot size) and direction (blue‐red color scale for lower to higher values) of associations between genus‐level microbial taxa and seizure frequency (primary outcome). (B) Bubble plot showing associations between genus‐level microbial taxa and the corresponding clinical variable (according to different colors) and the significance of the association (dot size). Positive effect size coefficients indicate that higher clinical index values are associated with increased taxon abundance, whereas negative coefficients indicate an inverse relationship, with taxon abundance decreasing as clinical index values increase.

### 3.4 Gut microbiome and severity indexes (secondary outcomes)

Longitudinal clinical indices were assessed and tested for associations with microbial taxa (Figure 5B; Supporting Information S6). Several genera showed nominal associations (*p* < 0.05) with CDD severity, including positive associations for *Prevotella, Parabacteroide*s, and *Fusicatenibacter*, and negative associations for *Faecalimonas* and *Mediterraneibacter*, though none remained significant after correction.

For gastrointestinal symptoms, SCFA‐producing genera (e.g., *Agathobacter, Gallintestinimicrobium*) were inversely associated with severity, while *Prevotella* and *Streptococcus* were positively associated; *Coprobacter* and *Hominenteromicrobium* remained significant after FDR correction (*q* ≈ 0.03).

Sleep disturbance and CGI scores showed similar patterns, with reduced abundance of SCFA‐producing taxa and enrichment of genera such as *Parabacteroides* and *Klebsiella*, although no associations survived correction. Overall, increased clinical severity was consistently linked to depletion of SCFA‐producing genera and enrichment of taxa associated with dysbiosis, whereas better developmental scores were associated with enrichment of anaerobic fermentative taxa and depletion of potentially pro‐inflammatory genera.

## 4 DISCUSSION

CDD is an early‐onset developmental and epileptic encephalopathy characterized by drug‐resistant seizures and multiple comorbidities that significantly impair the quality of life of patients and their families.^14^ In this study, we explored a holistic therapeutic approach targeting the microbiota–gut–brain axis, aiming not only to improve seizure control but also to positively affect clinically relevant features such as sleep disturbances, gastrointestinal symptoms, and overall disease severity.

The medical food supplementation demonstrated a favorable safety and tolerability profile in both children and adults with CDD. Most adverse events were mild to moderate gastrointestinal symptoms, although they ultimately led to discontinuation of the medical food in some cases.

Seizure control remains a major therapeutic goal in CDD. Recent advances include the introduction of treatments such as ganaxolone,^15^ highly purified cannabidiol,^16^ and fenfluramine,^17^ as well as the ketogenic diet, although the latter may worsen pre‐existing gastrointestinal symptoms.^18^

In our cohort, participants showed improvements in seizure frequency, clinical severity, sleep quality, and gastrointestinal symptoms, although these changes did not reach statistical significance. Monthly seizure frequency generally decreased over the course of the study, but responses varied markedly between individuals. Interestingly, individuals who responded during the first phase did not maintain the same degree of benefit later in the study, underscoring the marked heterogeneity of treatment response and the potential need for individualized therapeutic approaches.

A trend toward improvement was also observed in CDD Severity Scale scores, with substantial agreement between clinician assessments and caregiver reports. Although a universally accepted endpoint for clinical trials in CDD is still lacking, the scale appears to provide a useful indicator of overall disease burden.^7^

Consistent with previous reports,^19^, ^20^ gastrointestinal symptoms and sleep disturbances were highly prevalent in our cohort. Longitudinal observations suggested a reduction in gastrointestinal discomfort together with greater stability of sleep patterns over time. These aspects are particularly relevant from a caregiver perspective and may contribute meaningfully to quality of life.

We also explored the relationship between gut microbiota changes and clinical outcomes to identify microbial profiles potentially associated with responsiveness to supplementation, as well as biomarkers linked to disease severity. Clinical improvements coincided with detectable microbiota shifts, including increased alpha diversity, enrichment of taxa associated with beneficial metabolic functions, and reductions in both fecal and systemic SCFAs.

More specifically, the rise in richness‐based diversity indices may indicate the appearance of low‐abundance or previously undetected taxa, leading to broader microbial community representation. Since reduced microbial diversity has frequently been associated with ecosystem instability and altered metabolic activity in neurodevelopmental and epileptic disorders,^21^ the increased richness observed in our cohort could reflect an expansion of rare microbial populations rather than major redistribution among dominant taxa.

When the cohort was stratified by treatment response, responders tended to show increased *Faecalibacterium* and *Phocaeicola* and reduced *Bacteroides* and *Prevotella*, whereas non‐responders maintained a genus‐level profile more similar to baseline (t0). Such shifts are consistent with the effects of fermentable fibers, including inulin and FOS, which are known to reshape microbial community structure by selectively stimulating carbohydrate‐utilizing bacteria and promoting downstream metabolic interactions.^22^, ^23^ Indeed, inulin or FOS has been reported to increase SCFA production, particularly acetate and butyrate, while enriching taxa including *Faecalibacterium*, *Roseburia*, *Bifidobacterium, and Phocaeicola*.^24^, ^25^, ^26^, ^27^

The expansion of *Faecalibacterium* is of particular interest, given its established role as a butyrate‐producing genus involved in intestinal health, immune modulation, and gut–brain axis signalling.^28^, ^29^, ^30^

Its increased abundance has been associated with improved mucosal homeostasis and reduced inflammatory tone, potentially reflecting a shift toward a more functionally favorable microbial environment.^31^ *Phocaeicola*, a member of the *Bacteroides* group, contributes to complex carbohydrate metabolism, degrading dietary polysaccharides and host‐derived glycans, and participates in microbial cross‐feeding by generating intermediate metabolites that can support secondary fermenters, including butyrate‐producing bacteria.^32^, ^33^

Consistent with the study's primary endpoint, we identified associations between bacterial taxa and seizure frequency. After FDR correction, *Dialister, Agathobaculum*, and *Mediterraneibacter* were negatively associated with seizure burden, whereas *Veillonella* and *Enterococcus*_A showed positive associations. Notably, the negatively associated taxa were predominantly butyrate‐producing genera, supporting a potential role for microbiota‐derived metabolites in gut–brain homeostasis. *Dialister* ^2^, ^34^ has previously been linked to improved outcomes during ketogenic diet therapy, while *Agathobaculum* includes butyrate‐producing species with reported neuroprotective effects in experimental neurodegenerative models.^35^, ^36^

In contrast, taxa positively associated with seizure burden, including *Veillonella*, *Klebsiella*, *Enterococcus_A*, and *Enterobacter*, are commonly linked to dysbiosis and intestinal inflammation. *Veillonella* has also been reported to be enriched in children with cerebral palsy and comorbid epilepsy, suggesting a potential association with neurological disease severity.^37^

An overall reduction in CDD severity scale scores was observed between baseline (t0) and the final study visit (t4). Because the scale was assessed only at these two time points, this change reflects the overall clinical course during the intervention and cannot be attributed to either MF1 or MF2 individually. Higher severity scores (worst clinical picture) were associated with increased abundance of *Prevotella* and *Fusicatenibacter* and lower abundance of *Faecalibacterium* and *Clostridium_A*, suggesting differences in microbial fermentative balance.^38^ Enrichment of SCFA‐producing taxa may reflect a more efficient conversion of metabolic pro‐inflammatory intermediates, such as succinate, supporting mucosal and metabolic homeostasis.^39^

Despite the metagenomic indication of increased fermentative activity, fecal and circulating SCFA concentrations progressively declined during the intervention. SCFA concentrations in our cohort were within the upper range of values typically reported in fecal and systemic compartments.^13^, ^40^

These data should be interpreted with caution. Fecal SCFA levels reflect the balance between microbial production, intestinal absorption and utilization, and intestinal transit, rather than SCFA production alone.^40^, ^41^ Similarly, the parallel decrease in circulating SCFAs does not support attributing the lower fecal levels solely to increased intestinal absorption, as circulating concentrations are also affected by intestinal and hepatic metabolism and peripheral utilization.^42^

The decrease involved not only butyrate but also acetate, propionate, and BCFAs. BCFAs, such as isobutyrate and isovalerate, are mainly produced through proteolytic fermentation,^43^ and their parallel decrease may therefore reflect a broader change in microbial metabolic activity rather than simply the effect of NaB supplementation. Notably, this reduction occurred despite the enrichment of bacterial taxa typically associated with fermentative metabolism and SCFA production, and in the absence of dietary changes among participants. This apparent dissociation suggests that changes in microbial composition and measured metabolic output do not necessarily occur in parallel. Several non‐mutually exclusive mechanisms may account for this finding, including changes in microbial production and cross‐feeding,^44^ substrate availability, intestinal transit,^43^ epithelial uptake and utilization, and host metabolism. Since these processes were not directly assessed, the mechanisms underlying the observed changes in SCFAs remain unclear. Since these processes were not directly assessed, the mechanisms underlying the observed changes in SCFAs remain unclear.

Our study should be interpreted considering several limitations. The study used a single‐arm, open‐label design, with all participants receiving MF1 followed by MF2. This approach was chosen because CDKL5 deficiency disorder is a rare condition with a limited number of eligible participants. However, without randomization or a control group, the effects observed during the MF2 phase cannot be clearly separated from time‐related changes or residual effects of MF1. The small sample size reduced the statistical power of the analyses and likely contributed to the loss of significance for most associations after FDR correction. Therefore, the findings should be considered exploratory and require confirmation in future controlled studies. In addition, the duration of both the intervention and washout phases was also relatively short. As a result, slower or more gradual clinical effects may not have been fully captured, and potential carryover effects between study phases cannot be excluded. Finally, stool and DBS samples were collected in a home setting, which may have introduced some variability in sample handling, preservation, and overall quality.

Despite these limitations, the decentralized longitudinal design enabled repeated multi‐omics and clinical assessments in a geographically dispersed rare disease cohort, reducing participant burden and improving feasibility. The integration of clinical, microbiota, and metabolomic data provides a comprehensive view of treatment‐related changes and supports further evaluation of this intervention in larger multicenter studies.

Taken together, our findings support a shift from symptom‐based treatment toward biomarker‐guided interventions in CDD. Although confirmation in larger multicenter cohorts is required, the combination of longitudinal clinical assessment with microbiome and metabolomic profiling may enable identification of responder phenotypes, facilitate patient stratification, and provide objective biomarkers to monitor treatment efficacy, thereby paving the way for microbiome‐informed precision medicine in developmental and epileptic encephalopathies.

## AUTHOR CONTRIBUTIONS

EB and AV conceived the study. FT, EO, and MDM conducted the experimental work. MDM and VC performed the data analysis. IV and MNS were responsible for patient enrolment and follow‐up. EB, AV, MDM, and VC drafted the manuscript. All authors reviewed and approved the final version of the manuscript.

## CONFLICT OF INTEREST STATEMENT

The authors declare that the research was conducted in the absence of any commercial or financial relationships that could be construed as a potential conflict of interest.

## ETHICAL PUBLICATION STATEMENT

We confirm that we have read the Journal's position on issues involved in ethical publication and affirm that this report is consistent with those guidelines.

## Supporting information


**Table S1:** Clinical characteristics of CDKL5 subjects (*n* = 13). The table summarizes key clinical features observed, including epilepsy onset and type, body mass index classification, anti‐epileptic drug usage, seizure frequency, motor and behavioral impairments, as well as gastrointestinal symptoms.
**Table S2:** Dietary intake across study timepoints. Data are presented as mean ± standard deviation.
**Figure S3:** Fecal fatty acid concentrations across timepoints. Boxplots show the distribution of fecal short‐ and medium‐chain fatty acids measured at timepoints t0–t4. Baseline (t0, purple), MF1 nutritional intervention (t1, blue), first washout period (t2, green), MF2 nutritional intervention (t3, yellow), and second washout period (t4, red). Concentrations are expressed as nmol/mg feces. *p < 0.001.
**Figure S4:** Systemic fatty acid concentrations across timepoints. Boxplots show the distribution of systemic short‐ and medium‐chain fatty acids measured at timepoints t0–t4. Baseline (t0, purple), MF1 nutritional intervention (t1, blue), MF2 nutritional intervention (t3, yellow). Concentrations are expressed as ng/mL. *p.
**Figure S5:** Heatmap depicting changes in genus‐level taxon abundance across timepoints. For each taxon, variation was estimated as the difference in median abundance between consecutive paired timepoints. The right‐side cladogram provides an overview of taxa displaying similar patterns of abundance variation. To improve visualization, values were scaled by matrix column.
**Table S6:** Table reporting the magnitude and statistical significance of associations between genus level taxa and clinical outcomes, estimated using mixed‐effects regression models implemented in MaAsLin2. Coefficient values indicate the direction and magnitude of taxon abundance changes associated with increasing clinical outcome values, whereas *p*‐values indicate the statistical significance of each association. Only nominally significant taxa were retained, defined as taxa with *p* value < 0.05 and *q*‐value > 0.05.

## Data Availability

The data that support the findings of this study are openly available in NCBI Sequence Read Archive (SRA) at https://www.ncbi.nlm.nih.gov/sra, reference number SUB16206286.
