# Supplementary material for: Exploring medical food supplementation as an adjunct strategy for microbiota restoration and seizure reduction in CDKL5 deficiency disorder
Source: Epilepsia Open. 2026 Oct 13:10.1002/epi4.70369. Online ahead of print. doi: 10.1002/epi4.70369 (PMC13660742; doi:10.1002/epi4.70369)
Supplement: Supplementary file 1 [file EPI4-9999-0-s001.pdf]

**Table S1**- Clinical characteristics of CDKL5 subjects (n=13). The table summarizes key clinical features observed, including epilepsy onset and type, body mass index classification, anti-epileptic drug usage, seizure frequency, motor and behavioral impairments, as well as gastrointestinal symptoms.

| **Clinical feature** | | **Subjects with CDD (%)** |
|---|---|---|
| Epilepsy | | 13/13 (100%)* |
| Age, median (IQR) | | 12 (8.8 – 17.5) |
| BMI | | |
| | Underweight | 10/13 (76.9%) |
| | Normal | 2/13 (15.3%) |
| | Overweight | 1/13 (7.6%) |
| Age at seizure onset | | |
| | <3 months | 2/13 (15.3%) |
| | 3-6 months | 9/13 (69.2%) |
| | >6 months | 2/13 (15.3%) |
| Seizure type | | |
| | Spasms | 9/13 (69.2%) |
| | Tonic | 3/13 (23.1%) |
| | Myoclonic | 1/13 (7.6%) |
| | Atypical absence | 4/13 (30.7%) |
| | Generalized tonic-clonic | 8/13 (61.5%) |
| Antiseizure medications§ | | |
| | One drug | 1/13 (7.6%) |
| | Two drugs | 5/13 (38.4%) |
| | Three drugs or more | 7/13 (53.8%) |
| Seizure frequency | | |
| | Daily | 3/13 (23%) |
| | Weekly | 9/13 (69.2%) |
| | Monthly | 1/13 (7.6%) |
| Motor impairment | | |
| | Unable to sit | 2/13 (15.3%) |
| | Seated | 11/13 (84.6%) |
| | Walks with support | 8/13 (61.5%) |
| | Walks independently | 1/13 (7.6%) |
| Other comorbidities | | |
| | Stereotipies | 13/13 (100%) |
| | Sleep disorders | 11/13 (84.6%) |
| | Visual impairment | 4/13 (30.7%) |
| | Autonomic disturbances | 13/13 (100%) |
| RTT behavior questionnaire (RSBQ) | | |
| | <30 | 4/13 (30.7%) |
| | >30 | 9/13 (69.2%) |
| GISI scale | | |
| | <3 | 7/13 (53.8%) |
| | >3 | 6/13 (46.1%) |
| Bristol Stool Scale | | |
| | <3 | 6/13 (38.4%) |
| | 3-4 | 7/13 (53.8%) |
| | >4 | 0/13 (0%) |

**Table S2.** Dietary intake across study timepoints. Data are presented as mean ± standard deviation.

| Dietary intake | t0 | t1 | t2 | t3 | t4 | p value |
|---|---|---|---|---|---|---|
| Energy (Kcal) | 1405 ± 424.7 | 1441 ± 518.5 | 1539 ± 536.8 | 1382 ± 406.3 | 1286 ± 345.1 | 0.541 |
| Starch (g) | 33.5 ± 21.7 | 35.7 ± 22.1 | 47.2 ± 29.3 | 38.4 ± 31.7 | 48.5 ± 32.5 | 0.636 |
| Carbohydrates (g) | 308.1 ± 325.6 | 192.5 ± 90.4 | 216.8 ± 99.4 | 184.7 ± 70.8 | 185.9 ± 69.0 | 0.538 |
| Carbohydrates (%) | 52.9 ± 11.6 | 50.7 ± 9.4 | 52.9 ± 7.4 | 50.6 ± 6.6 | 54.2 ± 6.9 | 0.636 |
| Insoluble fibre (g) | 3.9 ± 2.5 | 3.7 ± 2.9 | 3.9 ± 2.9 | 3.1 ± 2.2 | 2.8 ± 1.5 | 0.801 |
| Soluble fibre (g) | 1.34 ± 0.6 | 1.4 ± 0.9 | 1.35 ± 0.6 | 1.5 ± 0.7 | 1.3 ± 0.7 | 0.636 |
| Total fibre (g) | 13.4 ± 4.2 | 14.3 ± 6.6 | 15.3 ± 9.5 | 15.7 ± 9.0 | 12.4 ± 7.5 | 0.636 |
| Fat (g) | 48.4 ± 16.1 | 57.30± 24.7 | 54.6 ± 19.8 | 51.4 ± 19.7 | 42.8 ± 13.1 | 0.541 |
| Fat (%) | 33.2± 10.5 | 36.2 ± 7.0 | 33.1 ± 7.1 | 33.8 ± 6.6 | 30.4 ± 3.9 | 0.636 |
| Protein (g) | 46.75± 11.7 | 46.7 ± 16.9 | 51.7 ± 18.3 | 50.9 ± 8.3 | 46.70± 8.4 | 0.636 |
| Protein (%) | 14.0 ± 3.2 | 13.7 ± 3.9 | 14.0 ± 3.7 | 15.9 ± 3.8 | 15.8 ± 4.8 | 0.538 |

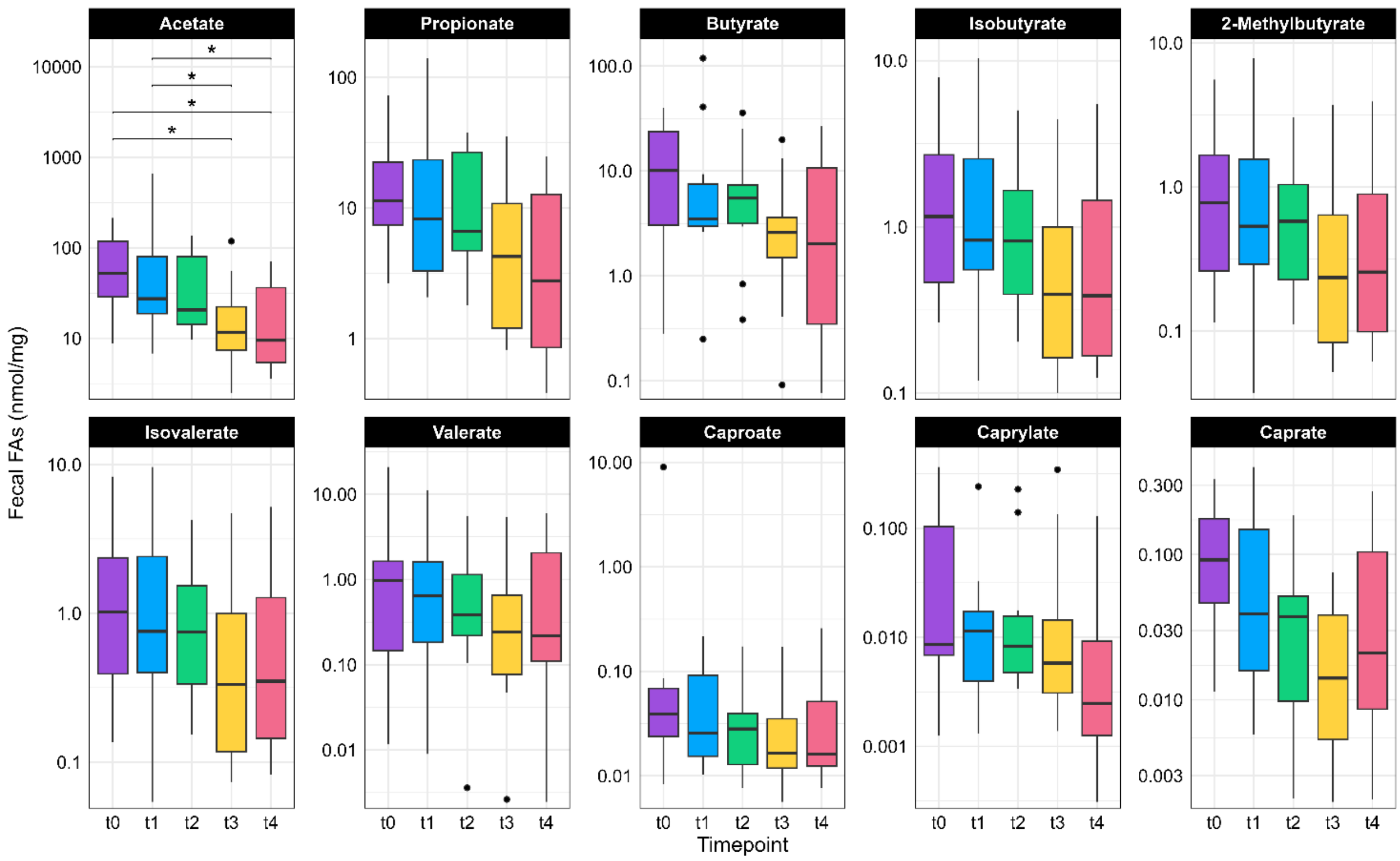


**Figure S3**. Fecal fatty acid concentrations across timepoints. Boxplots show the distribution of fecal short- and medium-chain fatty acids measured at timepoints t0–t4. Baseline (t0, purple), MF1 nutritional intervention (t1, blue), first washout period (t2, green), MF2 nutritional intervention (t3, yellow), and second washout period (t4, red). Concentrations are expressed as nmol/mg feces. * : p<0.05; ** : p<0.01; *** : p<0.001

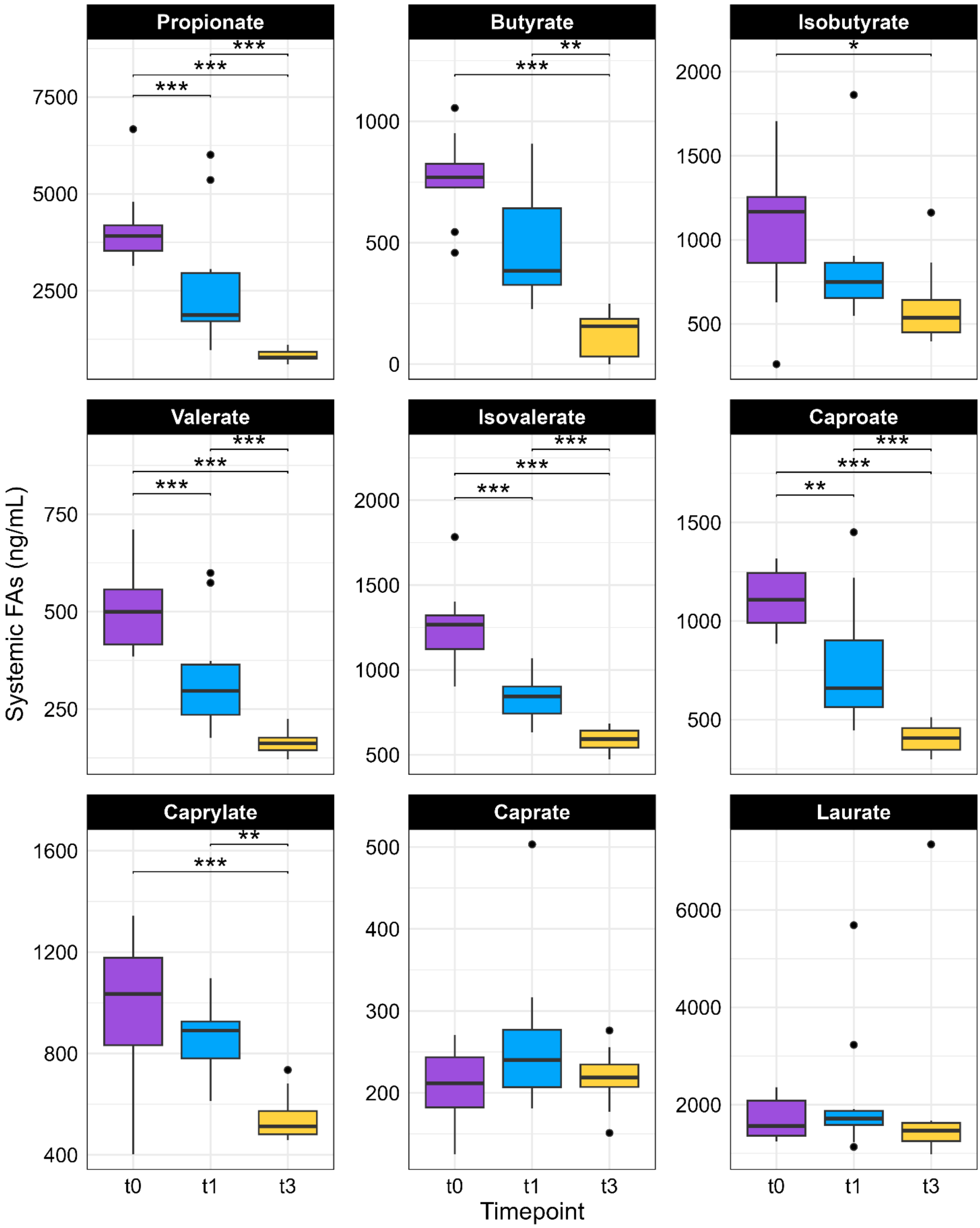


**Figure S4**. Systemic fatty acid concentrations across timepoints. Boxplots show the distribution of systemic short- and medium-chain fatty acids measured at timepoints t0–t4. Baseline (t0, purple), MF1 nutritional intervention (t1, blue), MF2 nutritional intervention (t3, yellow). Concentrations are expressed as ng/mL. * : p<0.05; ** : p<0.01; *** : p<0.001

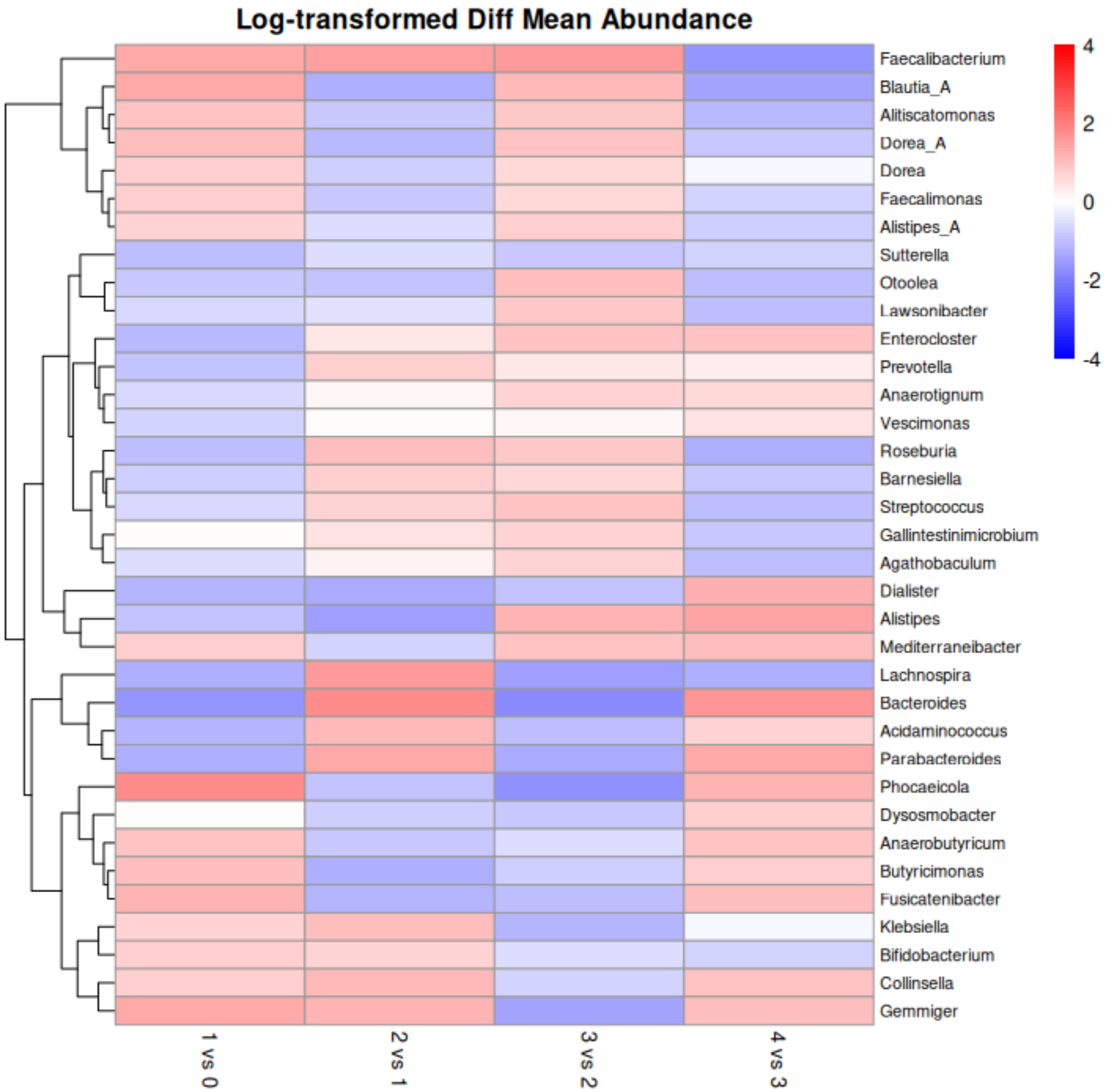


**Figure S5**. Heatmap depicting changes in genus-level taxon abundance across timepoints. For each taxon, variation was estimated as the difference in median abundance between consecutive paired timepoints. The right-side cladogram provides an overview of taxa displaying similar patterns of abundance variation. To improve visualization, values were scaled by matrix column.

**Table S6.** Table reporting the magnitude and statistical significance of associations between genus-level taxa and clinical outcomes, estimated using mixed-effects regression models implemented in MaAsLin2. Coefficient values indicate the direction and magnitude of taxon abundance changes associated with increasing clinical outcome values, whereas p-values indicate the statistical significance of each association. Only nominally significant taxa were retained, defined as taxa with p-value < 0.05 and q-value > 0.05.

| TAXON | value | coef | stderr | N | N.not.0 | pval | qval |
|---|---|---|---|---|---|---|---|
| *Bacteroides* | Sleep | -0.04 | 0.01 | 30 | 30 | 0.0007 | 0.0514 |
| *Coprobacter* | GISI | 0.54 | 0.14 | 30 | 12 | 0.0008 | 0.0381 |
| *Hominenteromicrobium* | GISI | 0.53 | 0.14 | 30 | 13 | 0.001 | 0.0381 |
| *Lawsonibacter* | RSBQ | 0.1 | 0.03 | 20 | 16 | 0.0028 | 0.2062 |
| *Parabacteroides* | CDD sev | 0.1 | 0.03 | 20 | 14 | 0.0036 | 0.2101 |
| *Odoribacter* | RSBQ | 0.16 | 0.05 | 20 | 10 | 0.0046 | 0.2062 |
| *Hominibacterium* | CDS | 1.21 | 0.37 | 20 | 8 | 0.0049 | 0.1473 |
| *Prevotella* | CDD sev | 0.31 | 0.1 | 20 | 13 | 0.0057 | 0.2101 |
| *Anaeromassilibacillus* | CDS | 1.35 | 0.44 | 20 | 8 | 0.0066 | 0.1473 |
| *Fusobacterium* | CDS | -0.82 | 0.26 | 20 | 7 | 0.0066 | 0.1473 |
| *Faecalimonas* | CDD sev | -0.25 | 0.08 | 20 | 12 | 0.0071 | 0.2101 |
| *Agathobaculum* | RSBQ | 0.14 | 0.05 | 20 | 17 | 0.0104 | 0.2974 |
| *Eisenbergiella* | CDS | 1.53 | 0.54 | 20 | 12 | 0.0112 | 0.1905 |
| *Streptococcus* | GISI | 0.46 | 0.17 | 30 | 24 | 0.0127 | 0.3289 |
| *Prevotella* | RSBQ | 0.19 | 0.07 | 20 | 13 | 0.014 | 0.2974 |
| *Dysosmobacter* | CDS | 1.1 | 0.41 | 20 | 16 | 0.0146 | 0.1905 |
| *Anaerotruncus* | CDS | 1.5 | 0.56 | 20 | 14 | 0.015 | 0.1905 |
| *Thomasclavelia* | RSBQ | -0.12 | 0.04 | 20 | 15 | 0.0167 | 0.2974 |
| *Parabacteroides* | Sleep | 0.05 | 0.02 | 30 | 21 | 0.0175 | 0.5183 |
| *Merdimmobilis* | Sleep | 0.08 | 0.03 | 30 | 11 | 0.0199 | 0.5183 |
| *Parabacteroides* | CGI | 0.6 | 0.24 | 20 | 14 | 0.0207 | 0.6766 |
| *Vescimonas* | CDS | 1.31 | 0.52 | 20 | 10 | 0.0218 | 0.242 |
| *Anaerostipes* | CGI | -1.19 | 0.47 | 20 | 18 | 0.0222 | 0.6766 |
| *Gallintestinimicrobium* | GISI | -0.43 | 0.18 | 30 | 12 | 0.0247 | 0.3883 |
| *Agathobacter* | MBA | -0.13 | 0.05 | 20 | 12 | 0.0251 | 0.6603 |
| *Klebsiella* | CGI | 1.53 | 0.63 | 20 | 10 | 0.0264 | 0.6766 |
| *Fusicatenibacter* | CDD sev | 0.18 | 0.07 | 20 | 16 | 0.0267 | 0.4126 |
| *Hominenteromicrobium* | CDS | 1.12 | 0.47 | 20 | 7 | 0.0269 | 0.2656 |
| *Agathobacter* | GISI | -0.6 | 0.26 | 30 | 17 | 0.0282 | 0.3883 |
| *Dialister* | MBA | -0.14 | 0.06 | 20 | 17 | 0.0288 | 0.6603 |
| *Mediterraneibacter* | CDD sev | -0.12 | 0.05 | 20 | 14 | 0.029 | 0.4126 |
| *Hungatella* | GISI | -0.36 | 0.16 | 30 | 19 | 0.0299 | 0.3883 |
| *Parabacteroides* | MBA | 0.05 | 0.02 | 20 | 14 | 0.032 | 0.6603 |
| *Coprobacillus* | CDD sev | -0.1 | 0.04 | 20 | 7 | 0.0332 | 0.4126 |
| *Bacteroides* | RSBQ | -0.03 | 0.01 | 20 | 20 | 0.0333 | 0.4518 |
| *Bacteroides* | CDS | -0.38 | 0.17 | 20 | 20 | 0.0357 | 0.2962 |
| *Clostridium_A* | CDD sev | -0.2 | 0.09 | 20 | 9 | 0.0359 | 0.4126 |
| *Clostridium_A* | MBA | -0.11 | 0.05 | 20 | 9 | 0.0367 | 0.6603 |
| *Flavonifractor* | CDD sev | -0.1 | 0.04 | 20 | 19 | 0.0371 | 0.4126 |
| *Collinsella* | CDS | 1.12 | 0.5 | 20 | 13 | 0.0381 | 0.2962 |
| *Clostridium_A* | CDS | 1.61 | 0.73 | 20 | 9 | 0.0399 | 0.2962 |
| *Eggerthella* | CDD sev | -0.13 | 0.06 | 20 | 15 | 0.0418 | 0.413 |
| *Prevotella* | GISI | 0.78 | 0.37 | 30 | 20 | 0.0433 | 0.4105 |
| *Sutterella* | GISI | 0.71 | 0.34 | 30 | 16 | 0.0441 | 0.4105 |
| *Mediterraneibacter* | MBA | -0.06 | 0.03 | 20 | 14 | 0.0442 | 0.6603 |
| *Mediterraneibacter* | CGI | -1.05 | 0.49 | 20 | 16 | 0.0443 | 0.6766 |
| *Hominiventricola* | RSBQ | 0.08 | 0.04 | 20 | 10 | 0.0453 | 0.4518 |
| *Lachnospira* | Sleep | 0.06 | 0.03 | 30 | 19 | 0.0458 | 0.7515 |
| *Agathobaculum* | CGI | -1.04 | 0.49 | 20 | 16 | 0.0465 | 0.6766 |
| *Lawsonibacter* | GISI | 0.28 | 0.13 | 30 | 24 | 0.0474 | 0.4105 |
